# Power of National Institutes of Health Stroke Scale in Assessing Stroke Systems of Care

**DOI:** 10.1161/JAHA.112.000521

**Published:** 2012-02-20

**Authors:** Edward C. Jauch

**Affiliations:** From the Division of Emergency Medicine, Department of Medicine, Medical University of South Carolina, Charleston, SC.

The timely article by Fonarow and colleagues in this issue of *JAHA* reports the findings from the largest study to date on the relation of the National Institutes of Health Stroke Scale (NIHSS) score with 30-day mortality rate in patients hospitalized for acute ischemic stroke.^1^ Prior smaller, largely single center, studies have reported similar results but this work used the largest data set to date with data from more than 400 hospitals involved in AHA “Get With The Guidelines—Stroke” program, representing more than 33,000 fee-for-service Medicare beneficiaries. The authors, using the initial NIHSS score alone as a continuous variable, demonstrated excellent discrimination of 30-day mortality rate, with a c statistic of 0.82. In this context, the c statistic provides a means for comparing the different prognostic models fitted to the same data and the best model is associated with the highest c statistic. The authors then compared their model to the reported Centers for Medicare and Medicaid Services (CMS) clinical Medicare risk-standardized mortality rate (RSMR) model for 30-day stroke mortality. The CMS RSMR for stroke is based on a hierarchical logistic regression model incorporating patient and hospital factors. The model adjusts for patient demographics, indicators of comorbidities, and disease severity, but not initial NIHSS score. The CMS model also adjusts for hospital-specific observed versus expected outcomes. Despite its complexity, the CMS RSMR model did not predict mortality as well as the initial NIHSS score alone (c statistic 0.82 versus 0.79, respectively).

Furthermore, categorical grouping of NIHSS score by risk-tree methodology into various tiers of stroke severity preserved the discriminatory capabilities with c statistics ranging from 0.74 to 0.80. Categorical grouping into 4 groups of increasing stroke severity by NIHSS score (low 0 to 7, medium 8 to 13, high 14 to 21, and very high 22 to 42) accurately predicted 30-day mortality rate nearly as well as when the full range of the NIHSS score was used (*c* statistic 0.80 versus 0.82, respectively), and again as well as or perhaps slightly better than the more complicated CMS models. Their results reinforce the importance of using the NIHSS score as a risk modifier in prognostic models used for stroke center certification, public reporting, and perhaps for pay-for-performance reimbursement in the future.

This publication comes at an important point in the organization of stroke systems of care. Organizations and governmental agencies in the United States and across the world are developing stroke systems of care as a way to improve patient outcomes after ischemic stroke.^2,3^ A stroke system of care is defined as a regional integration of stroke resources, including emergency medical services, emergency departments, hospitals, public health organizations, and advocacy organization into a singular stroke care delivery model. At the center of stroke systems of care are primary and comprehensive stroke centers.^4–5^ To justify the effort and expense of creating stroke systems of care and stroke centers, improvement in true patient-centered outcomes must be demonstrated. Data to verify that the creation of primary stroke centers has improved patient outcomes are just now forthcoming.^6–9^ Unlike primary stroke centers performance measures required by the joint commission and state certification programs, the AHA has recommended that comprehensive stroke centers report additional performance measures including patient outcomes for various interventions and forms of stroke, such as the “percentage of patients undergoing intracranial angioplasty and/or stenting for atherosclerotic disease with stroke or death within 30 days of the procedure.”10 Similarly the candidate performance measures for comprehensive stroke centers certification by the joint commission include both the initial NIHSS score and modified Rankin score at 30 days. This study highlights that in the determination of hospital-specific RSMR, the initial NIHSS score is a vital contributor to the prognostic power of the model. Future certification processes for primary stroke centers and comprehensive stroke centers may include an assessment of a RSMR as a requirement for reaccreditation.

The CMS began publicly reporting 30-day mortality rate for acute myocardial infarction and heart failure in 2007, for pneumonia in 2008, and intends to begin similar reporting of 30-day ischemic stroke RSMR. Concurrently, in an effort to in part improve quality and better empower healthcare consumers, CMS also intends to use in-house and 30-day mortality rate in their Medicare hospital value-based purchasing proposal.^11^ Currently stroke is not in the initial 2013 proposed clinical process of care measures for value-based purchasing, but stroke is likely to be addressed in the future. Using the current ischemic stroke RSMR models which do not include the initial NIHSS score may lead to unintentional financial disincentives in stroke centers which care for a disproportionate share of the most severe stroke patients. Given the current modest payment rates for stroke, this may lead to the unintended consequence of decreasing access to stroke expertise in stroke systems of care.

Public reporting of hospital-based mortality is not new but has become far more widespread over the past decade.^12^ Despite this experience, the effect of publicly reporting hospital performance does not ensure programmatic improvement in hospitals nor change healthcare consumer purchasing behaviors.^12–15^ Mortality is a poor measure of quality, especially for the lay public to use in making healthcare purchase decisions. Although mortality is a hard data point, actual measurements of quality of care are not well captured by themetric and the use of RSMR is often inappropriately used to directly compare two hospitals with one another. Because a stroke RSMR for individual hospitals will be reported for the foreseeable future, collecting the initial NIHSS score and incorporating it into RSMR providing the best adjusted measure will be key.

In this edition of *JAHA* the work by Fonarow and colleagues for the first time provides the stroke and healthcare community a large-scale validation of our personal experiences of the prognostic power of the initial NIHSS score. It also highlights the importance of collecting the initial NIHSS score for registry data sets as well as incorporating the NIHSS score into RSMR models. Although the overall improvement in predicting stroke mortality incorporating the initial NIHSS score may be seemingly modest, over the current RSMR models (*c* statistic 0.82 versus 0.79, respectively), utilization of a more accurate model will allow better recognition of true performance and provide better discrimination of the quality of care across hospitals.
